# Data on the Facebook marketing strategies used by fast-food chains in four Latin American countries during the COVID-19 lockdowns

**DOI:** 10.1186/s13104-021-05870-8

**Published:** 2021-12-20

**Authors:** Lucila Rozas, Luciana Castronuovo, Peter Busse, Sophia Mus, Joaquín Barnoya, Alejandra Garrón, María Victoria Tiscornia, Leila Guanieri

**Affiliations:** 1 Instituto de Investigación Científica, Grupo de Investigación en Comunicación y Salud, Universidad de Lima, 4600 Javier Prado Este Avenue, Tower A, 11th Floor, 15023 Lima, Peru; 2 Fundación Interamericana del Corazón Argentina (FIC-Argentina), Arévalo 2364—1 A, C1425DBR Buenos Aires, Argentina; 3 Instituto de Investigación Científica, Grupo de Investigación en Comunicación y Salud, Carrera de Comunicación, Universidad de Lima, 4600 Javier Prado Este Avenue, Tower A, 11th Floor, 15023 Lima, Peru; 4 Departamento de Investigación, Unidad de Cirugía Cardiovascular de Guatemala, 9a Av 8-00 zona 11, Ciudad de Guatemala, 01011 Guatemala; 5 Fundación Interamericana del Corazón—Bolivia, Mariscal Santa Cruz Avenue, La Primera Building, Tower B, 10th Floor, Office No. 7, La Paz, Bolivia

**Keywords:** Food marketing, Social media, Food environment, Children, Adolescents, COVID-19, Latin America

## Abstract

**Objectives:**

During the COVID-19 pandemic, most countries implemented lockdowns that motivated changes in the dietary patterns, physical activity, and body mass index (BMI) of consumers worldwide, as well as the emergence of new food marketing strategies in social media. We sought to design and validate a methodology for monitoring and evaluating the Facebook marketing strategies of multinational fast-food chains in response to the COVID-19 pandemic.

**Data description:**

We developed three datasets. First, a dataset with the Uniform Resource Locators (URLs) of 1015 Facebook posts of five fast-food chains present in Argentina, Bolivia, Guatemala, and Peru. Second, a dataset of 106 content-analyzed posts we used in a pilot to determine intercoder reliability using statistical tests. Third, a dataset of a final sample of the 1015 content-analyzed posts that we used to determine the variables most frequently used. Following a mixed-methods approach, we developed 29 variables that recorded general information, as well as the marketing strategies we identified in the posts, including 14 COVID-19 specific variables. These data should help to monitor the social media marketing strategies that fast-food chains have introduced during the COVID-19 lockdowns, thus providing initial evidence about how they could be contributing to an unhealthy food environment.

## Objective

The objective was to design and validate a methodology for monitoring and evaluating the COVID-19 related Facebook marketing strategies of five multinational fast-food chains (Burger King, Starbucks, KFC, TGI Fridays, and Subway) present in Argentina, Bolivia, Guatemala, and Peru. The pandemic context led governments to implement lockdowns that influenced changes in the Body Mass Index (BMI), dietary patterns, and physical activity of children and adolescents [1–6], as well as demanded new marketing strategies by the food industry [7–11]. Moreover, it may have contributed to the growing exposure of young consumers to online, food-related advertising [12].

Previous studies analyzed the online marketing strategies food and beverage brands have used, including those adapted to social media platforms like Facebook [13–20]. Other research studies evaluated the potential impact of online marketing, especially among the younger population [21, 22]. Nonetheless, there is still little knowledge about the social media marketing strategies that the fast-food industry used during the COVID-19 pandemic since lockdowns and social distancing measures interrupted and/or changed the patterns of food consumption outside the household.

These data are unique since they contain a sample of 1015 Facebook posts collected from the country-specific fan pages of the previously mentioned fast-food brands during COVID-19 lockdowns. We also include information about the validation of our methodological tools. This work can help researchers analyze and compare the Facebook marketing strategies fast-food brands have introduced during this period, thus providing initial evidence about how they could have been contributing to an unhealthy food environment. The tools from this study can also empower public officials to monitor the social media environment over time in order to identify the marketing strategies of fast food companies that appear to be detrimental among vulnerable populations.

## Data description

We stored three datasets and the complementary documentation in the open data repository of the Inter-University Consortium for Political and Social Research (openICPSR) [23]. The first is a dataset with the uniform resource locators (URLs) of 1015 Facebook posts of five fast-food chains present in four Latin American countries. Second, a dataset with 106 posts that two researchers coded simultaneously to determine interrater agreement. And third, a dataset with the final sample of the 1015 posts that we content-analyzed independently to determine the most used marketing techniques during the study period.

We restricted the period of data collection to one month before the start of the lockdown measures and three months after in each country: (1) Argentina: February 20–June 20th (lockdown started March 20); (2) Bolivia: February 22–June 22nd (lockdown started March 22nd); (3) Guatemala: February 16–June 16th (lockdown started March 16th); (4) Peru: February 16–June 16th (lockdown started March 16th).

We used a mixed-methods approach to develop the methodological tools that would allow us to examine COVID-19 related content. This entailed two phases. During phase one, we manually extracted the screenshots and URLs of a random sample of 180 Facebook posts published within the specified months (around nine per brand for each country) and we assigned them unique identification codes. We included posts in every format allowed by the social media site: video, graphics interchange format (GIF) image, static image (one or more), slider, and text. Then, two researchers analyzed simultaneously these posts following a qualitative content analysis method, which led to consolidating a qualitative analysis codebook (see Table 1, data file 4). Due to its inductive and emergent nature, this method was suitable for developing the new variables that capture COVID-19 related content.

**Table 1:** Overview of data files/data sets

Label	Name of data file/data set	File types (file extension)	Data repository and identifier (DOI or accession number)
Dataset 1	Data_Uniform Resource Locators _All_FoodMkt&COVID19	Comma separated values (.csv)	ICPSR—Interuniversity Consortium for Political and Social Research. https://doi.org/10.3886/E144061V2 [23]
Dataset 2	Coded_QuantiData_Pilot_FoodMkt&COVID19	Comma separated values (.csv)	ICPSR—Interuniversity Consortium for Political and Social Research. https://doi.org/10.3886/E144061V2 [23]
Dataset 3	Coded_QuantiData_All_FoodMkt&COVID19	Comma separated values (.csv)	ICPSR—Interuniversity Consortium for Political and Social Research. https://doi.org/10.3886/E144061V2 [23]
Data file 1	Guide for coding_Qualitative Analysis_Food Marketing&COVID-19	Microsoft Word Open XML format document file (.docx)	ICPSR—Interuniversity Consortium for Political and Social Research. https://doi.org/10.3886/E144061V2 [23]
Data file 2	Guide for collecting and uploading posts_Qualitative Analysis_ Food Marketing&COVID-19	Microsoft Word Open XML format document file (.docx)	ICPSR—Interuniversity Consortium for Political and Social Research. https://doi.org/10.3886/E144061V2 [23]
Data file 3	Qualitative Analysis Matrix_Food Marketing&COVID-19	Comma separated values (.csv)	ICPSR—Interuniversity Consortium for Political and Social Research. https://doi.org/10.3886/E144061V2 [23]
Data file 4	Qualitative Codebook_ Food Marketing&COVID-19	Plain text file (.txt)	ICPSR—Interuniversity Consortium for Political and Social Research. https://doi.org/10.3886/E144061V2 [23]
Data file 5	Quantitative Analysis Codebook_Food Marketing&COVID-19	Plain text file (.txt)	ICPSR—Interuniversity Consortium for Political and Social Research. https://doi.org/10.3886/E144061V2 [23]
Data file 6	Quantitative Analysis Coding Form_FoodMarketing&COVID-19 (SP)	Portable document format (.pdf)	ICPSR—Interuniversity Consortium for Political and Social Research. https://doi.org/10.3886/E144061V2 [23]
Data file 7	Research Protocol_FoodMKT&Covid-19	Plain text file (.txt)	ICPSR—Interuniversity Consortium for Political and Social Research. https://doi.org/10.3886/E144061V2 [23]
Data file 8	RSyntax_FoodMKT&Covid-19	R archive file (.r)	ICPSR—Interuniversity Consortium for Political and Social Research. https://doi.org/10.3886/E144061V2 [23]
Data file 9	RSyntax_FoodMKT&Covid-19_txt	Plain text file (.txt)	ICPSR—Interuniversity Consortium for Political and Social Research. https://doi.org/10.3886/E144061V2 [23]
Data file 10	Data Obtention and Tool Validation Process Flowcharts_FoodMKT&Covid-19	Portable document format (.pdf)	ICPSR—Interuniversity Consortium for Political and Social Research. https://doi.org/10.3886/E144061V2 [23]

For phase two, we collected the URLs of 1015 Facebook posts. This included the 180 posts we analyzed previously. After assigning unique identification codes to the entire sample, researchers coded the data using quantitative content analysis. For this, we developed a new codebook and a digital coding form (developed on Google Forms), based on the qualitative tool designed during phase one and on previous research [15, 24]. The codebook was piloted using a subsample (n  = 106, see Table 1, dataset 2). We used R software (version 4.1.0) to calculate Krippendorf’s alpha index, which allowed us to test intercoder agreement and determine the reliability of our variables (Table 1, data files 8 and 9). After adjusting this tool, we independently coded and analyzed the entire sample (n  = 1015). Then we ran descriptive statistics to determine the variables that fast-food chains used most frequently. This final step led to the consolidation of our codebook (Table 1, data file 5). The entire process is detailed in two flowcharts included in the repository (see Table 1, data file 10) [23].

## Limitations

The total sample of Facebook posts we collected for this study only includes those published by the selected brands during the four-month periods we specified before. Therefore, it does not provide a full picture of the evolution of the marketing techniques on Facebook or other social media platforms such as Instagram or Twitter for the duration of the COVID-19 pandemic, as they needed to adapt to a rapidly evolving context in which lockdown measures relaxed and tightened. This may also impact the comprehensiveness of our methodological tools, which could be subject to minor changes. Furthermore, given that Facebook posts include branded content under intellectual property rights, we were unable to include screenshots of the posts in our data deposit, as it is impossible to obtain the appropriate permissions from the fast-food companies. Instead, we have only included the URLs of each post we analyzed, which could produce data loss in the long term.

## Data Availability

The data described in this Data note can be freely and openly accessed on the openICPSR repository under Reference Number openicpsr-144061, https://doi.org/10.3886/E144061V2. Please see Table 1 and reference [23] for details and links to the data.

## Abbreviations

BMI: Body Mass Index
URL: Uniform resource locators
GIF: Graphics interchange format
